# Improvements on activated sludge settling and flocculation using biomass-based fly ash as activator

**DOI:** 10.1038/s41598-019-50879-6

**Published:** 2019-10-10

**Authors:** Xiaoqian Chen, Fangong Kong, Yingjuan Fu, Chuanling Si, Pedram Fatehi

**Affiliations:** 1 State Key Laboratory of Biobased Material and Green Papermaking, Qilu University of Technology (Shandong Academy of Sciences), Jinan, 250353 China; 2 Tianjin Key Laboratory of Pulp and Paper, Tianjin University of Science & Technology, Tianjin, 300457 China; 3 Green Processes Research Centre and Chemical Engineering Department, Lakehead University, 955 Oliver Road, Thunder Bay, ON P7B5E1 Canada

**Keywords:** Chemical engineering, Colloids, Polymers

## Abstract

Biomass-based fly ash and wastewater are undesired products of the pulping industry. Recently, the use of biomass-based fly ash as an adsorbent (i.e., a valued material) for constituents of wastewater effluents was reported. In this work, the settling performance and properties of activated sludge were studied in the presence of fly ash. Upon mixing, fly ash increased the zeta potential of the sludge from −31 mV to −28 mV, which was due to the release of cationic ions from fly ash in the sludge suspension. The sludge settling and its flocculation affinity were improved through the complexation of flocs and released cation ions from fly ash. The relationships between the protein/polysaccharide (PN/PS) ratio and the content of extracellular polymeric substances (EPS) as well as the ratio and the properties of the sludge flocs were determined. A correlation between the total loosely bound-EPS (LB-EPS) content and the effluent suspended solids (ESS) (Pearson’s coefficient, *r*_p_ = 0.83) was observed. The performance of sludge flocculation and settling were much more closely correlated with LB-EPS than with tightly bound EPS (TB-EPS). Scanning electron microscopy (SEM) analysis of sludge flocs before and after EPS extraction showed that the sludge flocs contained a large number of microorganisms, mainly Bacillus and Cocci. The amount of LB-EPS had an adverse influence on bioflocculation, effluent clarification and sludge settling affinity. The sludge properties had a moderate relationship with the PN/PS ratio of LB-EPS. Also, no correlation could be established between the ratio and the TB-EPS content.

## Introduction

The activated sludge process is one of the major techniques for treating industrial wastewater^1^. Even though most of the pollutants can be removed in the biological treatment process, there are still some nonbiodegradable organic substances that cannot be removed via a biological treatment. These substances can do a great damage to the environment^2^. Therefore, it is important to improve the efficiency of wastewater treatment systems for removing these substances. The addition of adsorbents to activated sludge has shown to improve the efficiency of the aerobic systems^3^. In this process, the porous structures and high surface areas of adsorbents help adsorb pollutants and bacteria^4^. Adsorption of pollutants would decrease pollutants’ inhibitory effects in the bulk solution, but it alters the characteristics of sludge^5,6^.

Biomass-based fly ash is generated as a byproduct of boilers incinerating forest residues, such as bark, wood chip and sawdust, and its major chemical components are aluminium oxide, silica oxide, ferric oxide, calcium oxide, magnesium oxide and carbon, which provides it with alkaline nature^7^. It is a relatively abundant and inexpensive material comparing with the other adsorbents, such as activated carbon or zeolites. Most of fly ash is disposed of in landfills as wastes, which is costly with major environmental footprints. These reasons provide incentives for identifying alternative uses for biomass-based fly ash. The size, the morphologies and chemical compositions of fly ash are similar to those of kaolin used as a filler in papermaking, making fly ash a promising sustainable material for papermaking operations (e.g., for corrugated medium papers, container boards)^8^. Alternatively, biomass fly ash was reported as an adsorbent for the removal of recalcitrant organic pollutants from pulping wastewater^9^. In the present study, biomass-based fly ash was integrated into the sequential batch reactor (SBR) as adsorbent and activator to improve the efficiency of the biological treatment. In our earlier work, the operation of an SBR for pulping wastewater was examined^10^. Fly ash supplementation improved effluent qualities and enhanced the settling ability of activated sludge. The objective of the research described in this work is to understand the mechanism of fly ash interaction with sludge.

The efficiency of biological processes highly depends on the metabolic capability of microorganisms and the physicochemical characteristics of activated sludge^11^. The extracellular polymeric substances (EPS) are the major components of the activated sludge flocs and mainly responsible for the physicochemical properties of the flocs including flocculation, sludge settling and sludge dewatering^12^. EPS originate from metabolism or lysis of microorganisms and contain polysaccharides (PSs), proteins (PNs), and nucleic acids. EPS is categorized into soluble and bound EPS, and bound EPS are closely bound with cells, while soluble EPS are weakly bound with cells or dissolved into wastewater solution^13^. Bound EPS include microbially produced polymers, lysis and hydrolysis products and are divided into loosely bound EPS (LB-EPS) and tightly bound EPS (TB-EPS)^14–16^. EPS is believed to be essential for sludge floc formation, but an increase in LB-EPS may weaken the floc structure and cell attachment, resulting in poor bioflocculation and sludge-water separation^17,18^. Li and coworker investigated the effect of LB-EPS on the flocculation and sedimentation of activated sludge, and the results showed that when the LB-EPS contents were higher than 5.5 mg/g TOC over mixed liquor suspended solid (MLSS), the effluent quality worsened with increasing effluent suspended solids (ESS)^17^. Some studies have suggested that the compositions and properties of EPS, rather than the quantity of EPS, are important in sludge flocs^19,20^. The effect of the main components of EPS on the settling ability and flocculation of sludge flocs in SBRs have not been well documented. The effect of the quantities and compositions of LB-EPS and TB-EPS on the sludge sedimentation and flocculation were investigated in this SBR system, and the variations in the EPS characteristics with the fly ash addition were further examined in this work. The second objective of this work was to study the correlation between the sludge properties and EPS characteristics, and to identify the effect of fly ash addition on the sludge and EPS characteristics. This is the first report on the interaction of biomass-based fly ash and activated sludge.

In this laboratory study, activated sludge was treated with biomass-based fly ash (as an adsorbent). The effluent quality and sludge-water separation was examined with and without fly ash addition. The microscopic structures and the components of activated sludge flocs were investigated. The respective effects of LB-EPS and TB-EPS on the sludge flocculation and sedimentation properties were also determined. Such information would be useful in understanding the role of fly ash in bioreactors, particularly in relationship with sludge flocculation and settling.

## Results and Discussion

### Characteristics of biomass-based fly ash

The characteristics of biomass-based fly ash used in this experiment are listed in Table 1. The surface area of fly ash was 63.70 m^2^/g and it contained 35.78 wt.% of unburned carbon, which made it a high-potential adsorbent. It contained 19.33 wt.% metals, mainly 10.35 wt.% calcium, 2.36 wt.% potassium and 2.29 wt.% aluminum. The metal content of fly ash would facilitate its use as a potential coagulant for sludge flocs^10^.

**Table 1:** Characteristics of biomass-based fly ash^10^.

Parameters	Value
Surface area, m^2^/g	63.70
C, wt.%	35.78
Al, wt.%	2.29
Ca, wt.%	10.35
Fe, wt.%	1.00
Mg, wt.%	1.11
K, wt.%	2.36
Na, wt.%	0.64

### Performance of the SBRs

**Figure 1:**
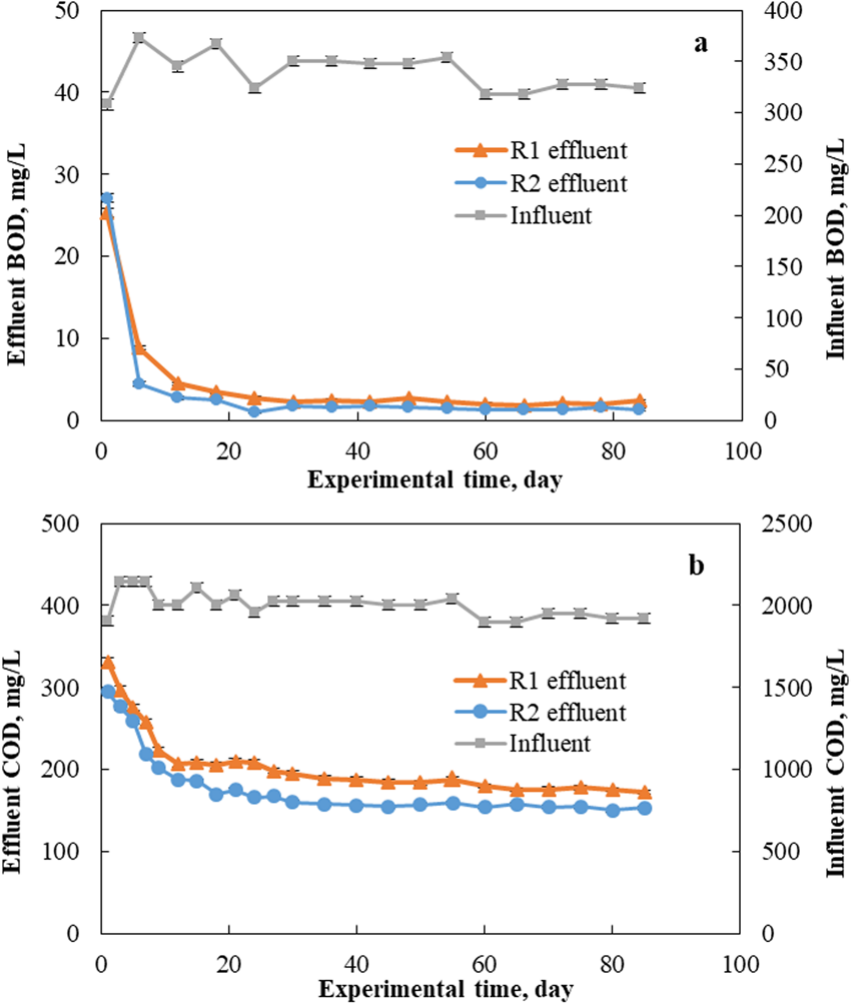
Variations in (**a**) BOD and (**b**) COD in influent and treated effluents. R1 functioned as a control reactor, and R2 functioned as the sample reactor with 0.5 g/L of fly ash dosage.

In this study, experiments lasted about three months and the steady state conditions for two SBR systems were generally reached one month after inoculation. Figure 1(a,b) show the changes of BOD and COD in the effluents. Irrespective of fly ash addition, the BOD in two reactors (1.24–2.68 mg/L) was relatively low. These results indicated that the biodegradable materials were almost completely removed by aeration with or without fly ash addition. However, there was still residual COD in the effluent for both reactors, and the residual COD in R1 was higher than that in R2 with significant differences (T-test, *P* < 0.05). Previous work claimed that fly ash addition improved an organic removal from SBR through the synergistic effect of adsorption and biodegradation^10^. The improved organic removal from R2 can be attributed to the increased adsorption of non-biodegradable and slowly biodegradable materials due to the presence of fly ash^21–23^.

**Figure 2:**
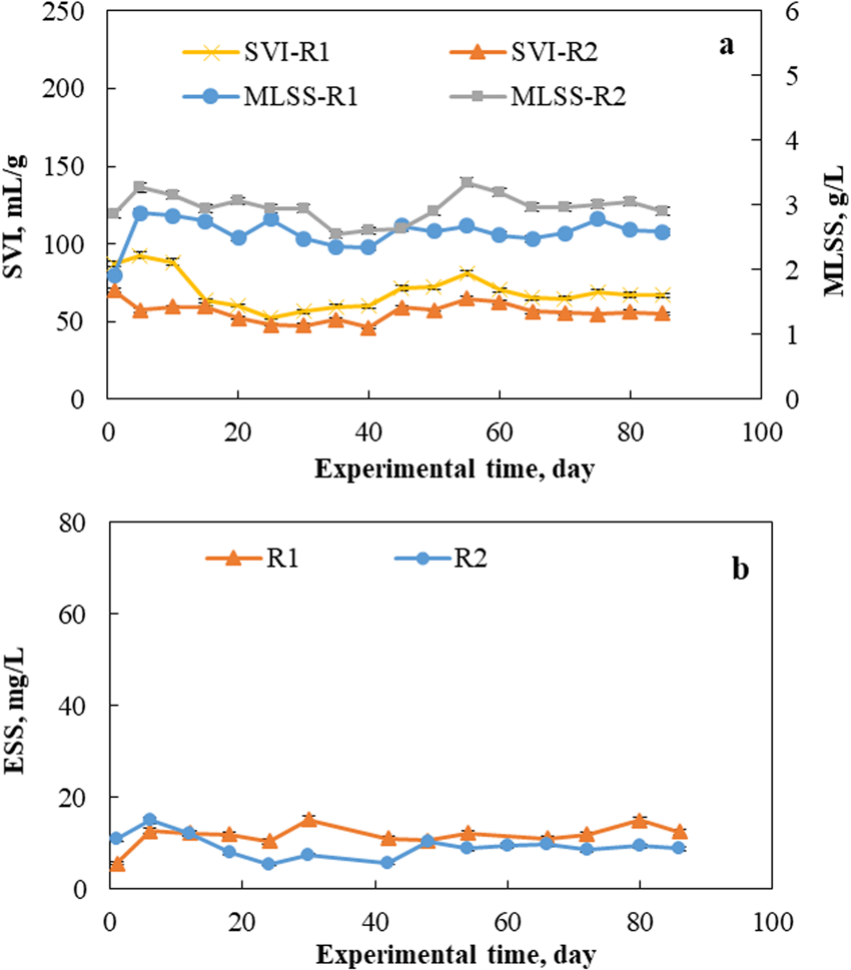
Variations in (**a**) SVI and MLSS and (**b**) ESS in treated effluent of two reactors. R1 functioned as a control reactor, and R2 functioned as the sample reactor with 0.5 g/L of fly ash dosage.

In the past, sludge volume index (SVI) and effluent suspended solid (ESS) were widely used for characterizing sludge settling and flocculation performance. Figure 2 shows the variations in the SVI, ESS and MLSS concentrations in the two reactors. It was observed that the MLSS of R2 was around 3 g/L. This MLSS contains 0.5 g/L of fly ash indicating that biomass sludge concentration was approximate 2.5 mg/L, which was consistent with the biomass content of R1. The ESS had no correlation with MLSS in this operation (*r*_*p*_ = −0.25 for R1 and *r*_*p*_ = 0.36 for R2 significant at 0.05 level), indicating limited dependence of ESS on the biomass concentration in SBRs. From Fig. 2a, a strong correlation between SVI and MLSS was observed after the 35th day in both two reactors (*r*_*p*_ = 0.73 for R1 and *r*_*p*_ = 0.77 for R2 significant at 0.05 level), indicating the negative effect of MLSS on SVI when the system reached the steady state condition. However, the SVI had the same variations with respect to MLSS in R1 and R2 implying that the MLSS did not specifically impacted either of the reactor. Therefore, the difference of SVI between R1 and R2 was mainly attributed to the presence of fly ash. The SVI in R1 varied between 56.45 and 80.87 mL/g, and the SVI in R2 varied between 45.98 and 64.89 mL/g. Although the ESS variation was marginal, the ESS of R2 effluent was slightly lower than the ESS of R1 effluent during the operation. These results demonstrated that the settling and flocculation performances of the sludge in R2 were better than those of sludge in the control reactor, R1, implying that the added fly ash in R2 played an important role in flocculating the sludge flocs. Previous studies claimed that there were multivalent mineral cations, such as calcium and magnesium, present on the surface of fly ash, and these cations bound with sludge flocs after their leaching, improving sludge flocculation and settling performance^10^. This was further confirmed by the increase in the zeta potential of the sludge suspension from −31 mV in R1 to −28 mV in R2. Therefore, lower SVI and ESS values indicated the positive impact of fly ash on sludge settling and flocculation properties. This finding was important in activated sludge systems, which was reported to reduce bulkiness and foaming challenge of sludge in industrial wastewater treatments^24^.

**Figure 3:**
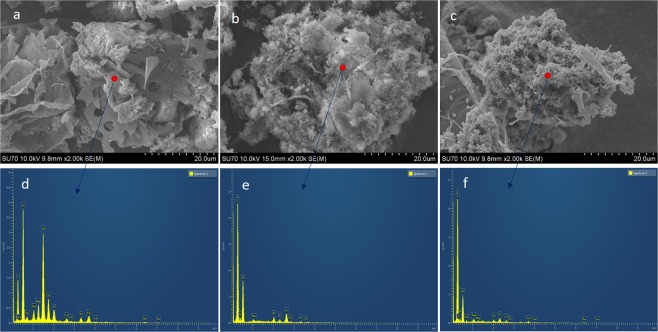
SEM morphology of (**a**) fly ash, (**b**) sludge flocs in R1, (**c**) sludge flocs in R2 and the energy-dispersive X-ray spectroscopy (EDX) analysis of the surface of (**d**) fly ash, (**e**) sludge flocs in R1, (**f**) sludge flocs in R2. R1 functioned as a control reactor, and R2 functioned as the sample reactor with 0.5 g/L of fly ash dosage.

Figure 3 illustrated the SEM morphology of fly ash and sludge floc in two systems. There were no filamentous bacteria observed in both reactors, indicating that both aerations were operated in good conditions^25^. It was evident from Fig. 3a that fly ash possessed an irregular shaped and porous structure. It was observed that the structure of sludge was fluffy and soft in the control reactor R1 (Fig. 3b), while a more rigid and compact structure was dominant in R2 (Fig. 3c). Moreover, the specific surface area of fly ash was 63.7 m^2^/g, while the surface areas of sludge in R1 and R2 were 5.9 m^2^/g and 2.5 m^2^/g, respectively. The smaller surface area of sludge in R2 indicated the more compact structure of the sludge flocs, and the attachment of sludge flocs to fly ash^5^. This was demonstrated in Fig. 3c with the apparent deposition of sludge particles on the fly ash particles in R2. The composition of the surface layer of fly ash and sludge flocs in R1 and R2 was analyzed using energy dispersive X-ray spectroscopy (EDX) (Fig. 3d–f). The dominant element of fly ash was carbon, oxygen, siliceous, aluminum, calcium and magnesium cations, which were observed in the surface of fly ash in Fig. 3d. Carbon, oxygen, nitrogen, sodium, phosphorus and sulfur were found in the surface of sludge flocs of R1 (Fig. 3e). Two different points were analyzed for the sludge flocs in R2. In addition to the elemental compositions observed on the surface of sludge flocs in R1, calcium and magnesium were detected in the surface of sludge flocs in R2 (Fig. 3f). These calcium and magnesium derived from fly ash and they were reported to play important roles in binding sludge flocs and making the sludge structure more rigid and compact in the aeration process^13^. It is inferred that the presence of calcium and magnesium in the fly ash of R2 contributed to the compact structure of sludge in R2 (Fig. 3c). This conclusion is consistent with the findings of Lin and coworkers in that the compactness of sludge flocs was enhanced through bridging between the flocs and cations ions^26^. In addition, the results in Fig. 3c,d confirmed the presence of fly ash in R2 and the attachment of sludge flocs in fly ash particles in R2 (Fig. 3c).

### Effect of fly ash on composition of EPS

Extracellular polymeric substances (EPS) of sludge flocs were extracted and measured weekly from the two reactors, and the average values are presented in Table 2. According to previous studies, PN was the principal component, and PS was the second component of the EPS matrices in activated sludge^16^. In the present study, PN, PS, PN/PS ratio and the total content of loosely bound LB-EPS, tightly bound TB-EPS and total EPS were analyzed. As tabulated in Table 2, the total content of LB-EPS decreased from 14.05 mg/g MLSS in control reactor R1 to 12.12 mg/g MLSS in R2, whereas the TB-EPS content showed no significant change in two reactors (2.05 mg/g MLSS for R1 and 1.96 mg/g MLSS for R2). This indicated that fly ash had more influence on LB-EPS than on TB-EPS. According to previous reports, LB-EPS played an unfavorable role in aerobic reaction, and fly ash appeared to reduce their effects^27^.

**Table 2:** Composition analysis of EPS extracted from sludge flocs in R1 and R2.

EPS	Compositions	Contents in R1	Contents in R2
LB-EPS	PN, mg/g	6.38	5.99
	PS, mg/g	7.67	6.13
	Total, mg/g	14.05	12.12
	PN/PS	0.83	0.98
TB-EPS	PN, mg/g	51.61	50.47
	PS, mg/g	25.19	25.75
	Total, mg/g	76.80	76.22
	PN/PS	2.05	1.96
Total-EPS	PN, mg/g	57.99	55.49
	PS, mg/g	32.86	31.78
	Total, mg/g	90.85	87.27
	PN/PS	1.76	1.75

Fly ash addition also changed the PN/PS ratio in EPS. For LB-EPS, the PN/PS ratio was higher in R2 (0.98) than in R1 (0.83). For TB-EPS, the changes were less significant. It was described that PNs were hydrophobic polymers, whereas PSs were hydrophilic polymers^28^. As demonstrated in previous studies, the hydrophilicity of EPS had a strong positive correlation with the adsorption affinity of fly ash^22^, thus it can be stated that fly ash adsorbed more amounts of PS than PN, which resulted in a higher PN/PS ratio in R2. Previously, a higher PN/PS was obtained due to the reduction of PSs by adsorbing to activated carbon^5^. Furthermore, the PNs had a high content of negatively charged amino acids^29^, which were involved more than PSs in binding with multivalent cations to stabilize the aggregate structure of sludge flocs^30^. Additionally, a significantly higher PN/PS ratio was observed for TB-ESP (1.96–2.05) than for LB-EPS (0.83–0.98) in both reactors, implying that more TB-EPS and less LB-EPS would increase the PN/PS ratio in the total EPS. In summary, fly ash addition decreased the content of LB-EPS and increased the PN/PS ratio in LB-EPS.

### Relationships between EPS and sludge settling and flocculation performance

Several studies demonstrated EPS to have a negative effect on the settling affinity and flocculation of activated sludge flocs^15,17,31^. A statistical analysis of the correlations between EPS properties (PN content, PS content, total content and PN/PS ratio) and sludge settling affinity, measured as SVI and flocculation, measured as ESS, were conducted, and the results are tabulated in Table 3. It was found that PN, PS and the total content of LB-EPS were positively correlated with the SVI and ESS (Table 3). The high concentration of these polymers measured in LB-EPS were related to their high SVI and ESS. Interestingly, the PN/PS ratio in LB-EPS had a strong negative correlation with SVI and ESS.

**Table 3:** Summary of Pearson’s correlation coefficient (*r*_p_) and *P*-value between the EPS components and the SVI and ESS.

EPS components		SVI		ESS	
		*r*_p_	*P*	*r*_p_	*P*
LB-EPS	PN	0.24	0.001	0.47	0.001
	PS	0.70	0.001	0.89	0.001
	Total	0.59	0.001	0.83	0.001
	PN/PS	−0.57	0.001	−0.53	0.001
TB-EPS	PN	0.20	0.001	0.09	0.000
	PS	0.08	0.001	0.13	0.025
	Total	0.14	0.001	0.14	0.001
	PN/PS	−0.02	0.001	−0.13	0.001

**Figure 4:**
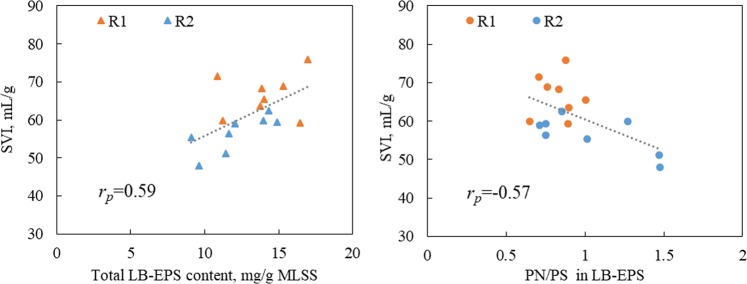
The relationships between the total content of LB-EPS and the PN/PS ratio in LB-EPS and the sludge settling ability as measured by the SVI. R1 functioned as a control reactor, and R2 functioned as the sample reactor with 0.5 g/L of fly ash dosage.

Statistical analysis indicated that the total content of LB-EPS (0.59) and PN/PS ratio in LB-EPS (−0.57) demonstrated modest correlations with the SVI. However, no significant correlation existed between these parameters and SVI in TB-EPS. Although the quantity of TB-EPS was greater than that of LB-EPS (Table 2), it was LB-EPS that functioned as the primary surface for cell interaction and attachment. Therefore, LB-EPS had a more significant effect than TB-EPS on the flocs’ structure. The relationships between the total content of LB-EPS and the PN/PS ratio and the SVI of the sludge flocs in R1 and R2 are illustrated in Fig. 4. It demonstrated that the presence of a large LB-EPS had a negative effect on settling performance of the activated sludge, and the PN/PS ratio in LB-EPS had a positive effect on the sludge’ settling characteristics, which was consistent with the observation that a lower content of LB-EPS and a higher PN/PS ratio of LB-EPS in R2 resulted in lower SVI, i.e., better settle ability.

The flocculation affinity of sludge flocs is an important character for turbidity removals and high effluent qualities in wastewater treatment^16^. The interaction between EPS and cells have a significant effect on sludge flocculation ability. As EPS were biopolymers that surround the bacterial cells, substrate must pass from wastewater through the EPS layer for transferring to cells, and thus a high level of EPS was not beneficial for substrate’s mass transfer^32^. In Liu and Fang’s study, EPS also had adhesion nature and acted as glue in maintaining cells together^15^. It was reported that a higher EPS content corresponded to higher flocculation^29^. Hence, the relationship between EPS and sludge flocculation was complex under different conditions.

The significant positive correlation between the total LB-EPS content and the ESS (*r*_*p*_ = 0.83) was observed, while no correlation between TB-EPS content and ESS was observed in Table 3, indicating that the performance of sludge flocculation was much more closely correlated with the amount of LB-EPS than with the amount of TB-EPS, and higher LB-EPS contents corresponded to higher ESS values. This was because the dispersible particles were loosely glued by LB-EPS, and these particles would be readily detached from floc surface and became dispersed, resulting in an increase in the suspension’s turbidity^33^. A similar result was found in a previous work that LB-EPS had a relatively weak capacity to bind the sludge flocs, and a high level of LB-EPS resulted in an ESS increase^27^.

**Figure 5:**
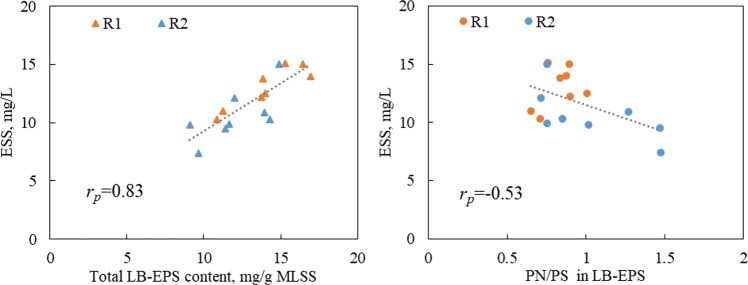
The relationships between the total content of LB-EPS and the PN/PS ratio in LB-EPS and the sludge flocculation as measured by the ESS. R1 functioned as a control reactor, and R2 functioned as the sample reactor with 0.5 g/L of fly ash dosage.

It was reported that PN and PS were important components contributing to the flocculation of sludge flocs^29^. From Table 3, PN/PS ratio had a modest negative correlation (*r*_*p*_ = −0.53) with ESS in that a high PN/PS ratio resulted in a lower ESS value and better flocculation performance. Work reviewed by Liu and Fang indicated that EPS were bound with cells mainly through ion bridging with multivalent metals, and these metals preferentially bound with PN rather than with PS^15^. This is consistent with other studies describing an excess amount of PS in the sludge flocs leading to poor effluent quality and poor flocculation^34^. Furthermore, the hydrophobic property of PN was also attributed to the separation of sludge and effluent^35^. It is concluded that the lower LB-EPS content and higher PN/PS ratio in LB-EPS resulted in lower ESS value, i.e., better flocculation of sludge flocs. The relationships between the total content of LB-EPS and the PN/PS ratio and the flocculation of the sludge flocs (ESS) in R1 and R2 are illustrated in Fig. 5. It can be seen that a lower LB-EPS content and higher PN/PS ratio were mostly obtained from sludge in R2, which correlated well with the better flocculation performance in R2.

### SEM images of sludge flocs before and after EPS extraction

**Figure 6:**
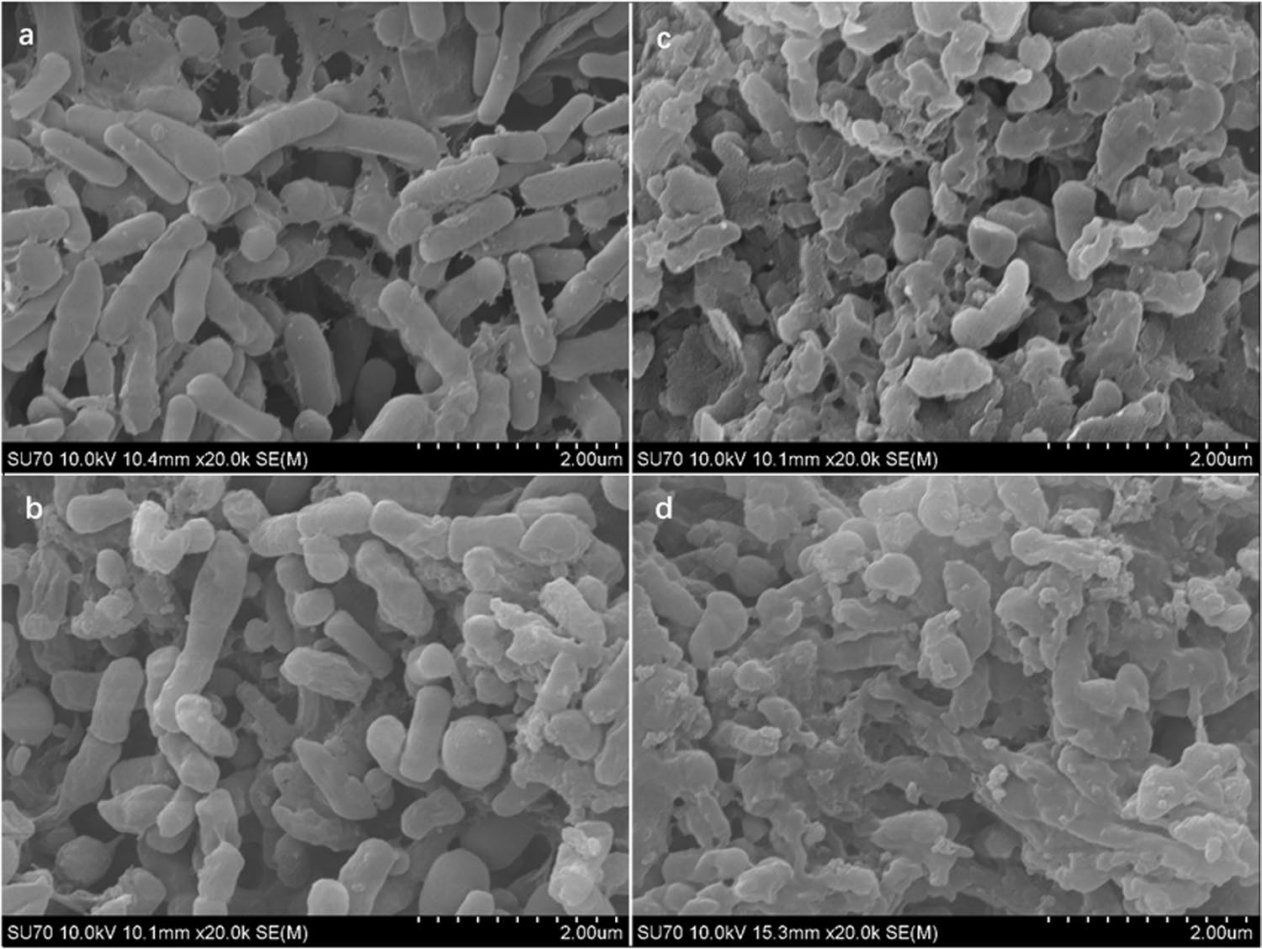
SEM images of the surface of sludge flocs before EPS extraction in R1 (**a,b**) R2 and after EPS extraction in (**c**) R1 and (**d**) R2. R1 functioned as a control reactor, and R2 functioned as the sample reactor with 0.5 g/L of fly ash dosage.

Figure 6 shows the surface structure of sludge flocs before and after EPS extraction. Figure 6a depicted that the floc of raw sludge in R1 contained a large number of microorganisms, mainly bacillus and cocci^36^. The sticky EPS surrounded the bacteria cells and linked the microbial communities to form the complex and stable reticular structures^37^. A porous structure and large specific surface area were observed in R1, resulting in the loose structure and high hygroscopicity, which were in favor of settling and dewatering of sludge flocs. There was a slight variation in the structure of sludge flocs in R2 as the Fig. 6b displayed. Cocci and bacillus could still be found, while the porosity was reduced compared with the raw sludge in R1, which contributed to the formation of the more compact and dense structure of the flocs. In addition, the reduction of specific surface area contributed to the reduction of water adsorption, which was beneficial for the separation of water and sludge. Thus, the sludge settling and dewatering performances were improved via fly ash addition.

After the EPS extraction, the originally plump and smooth surface of the microspheres both in R1 and R2 became coarse and condensed as shown in Fig. 6c,d. Microbial aggregates tend to be unstable after the removal of their surface EPS^38^. This indicated that EPS had an important impact on the stability and flocculation of the flocs. This was mainly attributed to the TB-EPS rather than LB-EPS, as TB-EPS located in the inner layer of the bacterial cells and was the main constructive materials of microbial aggregates. These results are consistent with the previous report that sludge cells were glued by TB-EPS, and removing TB-ESP resulted in floc de-flocculation^33^. Additionally, it should be noted that too much EPS production can result in viscous bulking, which might lead to the reactor’s poor performance^4^.

## Conclusions

This study examined the effect of fly ash supplementation on sludge properties in SBRs treating pulping wastewater. The COD and BOD removals were improved because of the adsorption of non-biodegradable and slowly biodegradable materials to fly ash, and the compactness of sludge flocs was enhanced through bridging the flocs and the cations ions from fly ash. The amount of LB-EPS had a negative effect on bioflocculation, effluent clarification and sludge settling ability, and the sludge properties had a moderate positive correlation with the PN/PS ratio of LB-EPS, but no correlation could be established with the TB-EPS content. Addition of fly ash decreased the LB-EPS and increased the PN/PS ratio of LB-EPS. SEM analysis showed that the sludge floc with less porous and highly compacted structure was obtained in R2. This process is environmentally friendly and cost-effective, as it realizes the recycling of an industrial waste. These experimental findings demonstrated a novel, reliable and effective technology for the utilization of biomass-based fly ash. This process concept can be further applied in the full scale aerobic biological treatment systems and generally in municipal and industrial wastewater treatment facilities.

## Materials and Methods

### Materials

The influents used in this work were produced from a hot water treatment of hardwood chips at 80 °C for 1 h before a thermomechanical pulping process, and they were received from a pulping company in Northern Ontario, Canada. Fly ash was collected from the bark boiler of the same pulping company, in which wastewater sludges, sawdust and barks from softwood and hardwood species were burned for heat generation, and fly ash was produced as a by-product. Activated sludge was obtained from the secondary wastewater treatment system of the same pulping company. Potassium dihydrogen phosphate (KH_2_PO_4_), ammonium chloride (NH_4_Cl), sodium hydroxide (NaOH, >99.0%) and sodium chloride (NaCl) were obtained from Sigma-Aldrich Inc., USA and sodium hydroxide was diluted to 1 mol/L prior to use. The protein standard bovine serum albumin and polysaccharides standard glucose were purchased from Sigma-Aldrich company, USA. The protein assay kit including Lowry reagent and folin-ciocalteu reagent were purchased from Thermo Fisher Scientific Inc., USA. The chemical oxygen demand (COD) kits (K-7365) were purchased from CHEMetrix Inc., USA. The biological oxygen demand (BOD) nutrient buffer solution was purchased from HACH Company, USA.

### Sequential batch reactors (SBRs)

In this study, a 0.5 g/L dosage of fly ash was prepared in a bench-scale aerobic sequential batch reactor (SBR) for treating the pulping wastewater. This dosage was selected based on the fact that we noticed a marginal effect of dosage on the effluent quality and sludge characteristics in a previous study^39^. In this work, we did not intend to overdose the system. Therefore, lower dosage of fly ash (0.5 g/L) was investigated in this research. The reactor was made of a plexiglass pipe with a working volume of 2 L. A magnetic stirrer was provided to maintain sludge in suspension. Aeration was supplied in the reaction stage by aeration pumps. Two reactors were run for 90 days. Reactor 1, R1, functioned as a control reactor, whereas reactor 2, R2, operated by dosing 0.5 g/L of fly ash. For R2, 0.5 g of fly ash was mixed with 1 L of wastewater, and then the mixture was inoculated in the reactor together with 0.25 L of seeding sludge on the first day. Based on this analysis, the concentration of fly ash in the mixed liquor was 0.4 g/L. Approximately, 100 mL of mixed liquor was removed at the end of one experimental cycle. Therefore, 0.04 g of fly ash was added to the reactor with the influents to compensate for the loss of fly ash that was removed with mixed liquor in the beginning of every cycle so that the concentration of fly ash remained at 0.5 g/L (based on wastewater) all the time in the R2 reactor. The temperature was kept at 25 °C; the dissolved oxygen values maintained higher than 1 mg/L and pH values were controlled around 7 by adding NaOH^19^. The sludge age (solid retention time, SRT) was around 10 days for two reactors. The total time for one cycle of biological reaction was 12 h consisting of 10 min of fill, 11 h of aeration, 40 min to settle and 10 min for withdrawal. After sedimentation, the effluents discharged through the drain of the reactors and the effluent samples were collected for COD and BOD analyses. The seeding sludge had a concentration of 12.5 g/L, and an initial mixed liquor suspended solids (MLSS) concentration of of 2500 mg/L was obtained in the reactor after inoculation. The MLSS content maintained about 2500 mg/L by discharging sludge from the mixed liquor at the end of each cycle. The chemical compositions of the pulping wastewater and operational conditions of the SBR system are shown in Tables 4 and 5, respectively.

**Table 4:** The compositions of the pulping wastewater.

Parameter	Concentrations
COD, mg/L	2012
BOD, mg/L	339
Total nitrogen, mg/L	8.5
Total phosphorus, mg/L	1.8
Suspend solid, mg/L	77
pH	5.36

**Table 5:** Operating conditions of the SBR.

Parameters	Value
Cycle length, h	12
Solids retention time, day	10–12
Organic loading (kg BOD/m^3^d)	0.34
Operating temperature, °C	25
Dissolved oxygen, ppm	>1
Operational pH	7–8

### Effluent quality measurements

The COD of influents and effluents was determined according to the standard methods 5220 D^40^. In this analysis, 2 mL of samples were added into the COD kits, and the kits were incubated at 150 °C for 2 h and then the absorbency of the sample for UV was determined at 620 nm after 30 min of cooling by a UV/VIS spectrophotometer (Genesys 10S, Thermo Scientific Inc., USA) using a calibration curve^41^. The BOD of influents and effluents was determined according to the concentration gradient of dissolved oxygen before and after the incubation of samples and nutrient buffered solutions at 20 °C for 5 days^42^.

### Extracellular polymeric substances (EPS) extraction and analysis

The EPSs were extracted from sludge suspension in R1 and R2, respectively, every ten experimental days, and a two-step heat extraction method was adapted to extract the LB-EPS and TB-EPS from sludge samples^27,43^. The sludge suspension was dewatered by centrifugation in a 50 mL tube at 4000 g for 5 min. The supernatant was collected for water quality analysis. The precipitated sludge in the centrifuge tubes was collected and then resuspended into 0.05 w/v% NaCl solution to its original volume of 50 mL at 50 °C and immediately remixed by a vortex mixer (Fisher Scientific) for 1 min. The sludge suspension was centrifuged again at 4000 g for 10 min, and organic matter in the supernatant was considered as the mass of LB-EPS sludge. For the extraction of the TB-EPS, the sludge in the centrifuge tubes was resuspended again in 50 mL of 0.05 w/v% NaCl solution. The sludge suspension was heated to 60 °C in a water bath for 30 min, and then was centrifuged at 4000 g for 15 min. The organic material obtained in the supernatant liquid was regarded as the TB-EPS^27,43^. Both the LB-EPS and TB-EPS extractions were analyzed for PN and PS. The sum of the amounts of total PN and PS was considered to represent the total amount of LB-EPS or TB-EPS.

The PN content of the samples was analyzed using protein assay kit following the Lowry method with bovine serum albumin as the standards^44^. In this set of experiments, 50 µL of samples and 1 mL of the Lowry reagent were added to a bottle and the bottle was mixed well and then incubated at room temperature for 10 min. Afterward, 100 µL of folin-ciocalteu reagent were added to the bottle. The bottle was mixed well and then incubated at room temperature for another 30 min. The samples were tested by a UV/VIS spectrophotometer (Genesys 10S, Thermo Scientific Inc., USA) at 750 nm and the PN concentration was calculated using a standard curve^45^.

The concentration of PS in the samples was determined using an ion chromatography unit equipped with a CarboPac^TM^ SA10 column (Dionex Corporation, Canada) and a Thermo Scientific Electrochemical detector at 30 °C. The eluent was 1 mM of KOH and the flow rate was 1.2 mL/min. Samples were hydrolyzed with 4 wt.% sulfuric acid at 121 °C in oil bath (AC200, Thermo Fisher Scientific Inc., USA) prior to the ion chromatography analysis^46^.

### Physicochemical characteristics of the sludge

Sludge suspensions were sampled daily from the reactors. The mixed liquor suspended solids (MLSS), sludge volume index (SVI) and effluent suspended solids (ESS) of a sludge sample were measured in accordance with the standard methods 2710 D^40^. The MLSS was obtained by filtering the mixed liquor through a glass fiber filter and measuring the mass of the filter residue after drying at 105 °C for 24 h^47^. Also, 100 mL of sludge suspension were transferred to 100 mL cylinders and settled for 30 min, and the volume of precipitated sludge was recorded for calculating SVI. The SVI value specifies the sludge settle ability and compressibility^17^. The supernatant fraction was collected from top of these cylinders as the effluent, and their effluent suspended solids (ESS) were measured. The total amount of ESS in effluents was determined by filtering the effluents through a glass fiber filter and measuring the mass of the filter residue after drying at 105 °C for 24 h^47^. The ESS indicates the performance of the microbial flocculation and effluent clarification^27^.

The micrographs of sludge flocs were taken using a scanning electron microscope (SEM) (Hitachi S-570, Japan). Sludge samples were taken out from the reactors and were fixed in phosphate buffer (pH 7.0) with 2.5% glutaraldehyde for 12 h at 4 °C. The fixed samples were then washed with buffer three times (10 min for each washing series). Then, samples were dehydrated gradually by successive immersions in ethanol solutions of increasing concentrations (50%, 70%, 80%, 90% and three rounds of 100%)^48^. The dried samples were coated with gold prior to SEM analysis with an Emitech K550 sputter coater. All images were acquired digitally using Quartz PCI software (Vancouver, BC, Canada). The element components (e.g., C, O, Na, Mg, Al, S, Si, P, K, Ca and Fe) of sludge were detected using an energy dispersive X-ray (EDX) spectroscopy (JEOL JSM 5900 LV) coupled with the SEM^26^.

The specific surface area of sludge and fly ash were measured via nitrogen adsorption/desorption isotherms. In this method, fly ash was pretreated at 105 °C overnight for contamination removal prior to analysis. The sludge samples were taken out from reactors at the 60^th^ and 80^th^ days directly, and then ground into powders after freeze-drying treatment.The Brunauer-Emmett-Teller (BET) surface area of the fly ash and sludge samples was measured using a NOVA-2200e Autosorb according to a previously established method^49^.

### Elemental analysis

The organic carbon compound of the fly ash was determined using a Vario EL cube instrument (Germany). Fly ash was pretreated at 105 °C overnight and ground into powder before the detection. Then, 0.02 g of dried samples were transferred into the carousel chamber of the elemental analyzer and combusted at 1200 °C, and the generated gasses were reduced to analyze carbon contents. The metal elements of fly ash were measured directly by an inductive coupled plasma emission spectrometer (ICP), a Varian Vista Pro CCD (Canada) equipped with CETAC ASX-510 autosampler, a cyclonic spray chamber and a Seaspray nebulizer according to the method established in the past^46^.

### Statistical analysis

T-test statistical analysis was used to determine if two sets of data were significantly different from each other. This analysis was performed for determining the influence of fly ash on the COD, BOD, SVI and ESS in this work. The Pearson’s product momentum correlation coefficient (also referred to as Pearson’s coefficient, *r*_*p*_) was used to estimate the linear relationships between two parameters. The Pearson’s coefficient, *r*_*p*_, has a value between +1 and −1, where 1 is total positive linear correlation, 0 is nonlinear correlation, and −1 is total negative linear correlation. This analysis was performed to test the linear relationships between EPS properties (PS content, PN content, PN/PS ratio and total content of LB-EPS and TB-EPS) and SVI and ESS. The significance of correlation was established at a 95% confidence level (*P* value < 0.05). All statistical analyses were conducted with Statistica Software (Statsoft, Tulsa, OK, USA) on a PC computer^19,34^.

## References

[1] Meerbergen K (2018). Decolorization of reactive azo dyes using a sequential chemical and activated sludge treatment. J. Biosci. Bioeng..

[2] Guo J (2018). Fermentation and kinetics characteristics of a bioflocculant from potato starch wastewater and its application. Sci. Rep..

[3] Zhang B, Li Y, Li S, Li G, Sun Q (2018). Effect of inoculated and uninoculated aeration pretreatment on nutrients and phytotoxicity of anaerobic digestion effluent. Sci. Rep..

[4] Shao Y, Shi Y, Mohammed A, Liu Y (2017). Wastewater ammonia removal using an integrated fixed-film activated sludge-sequencing batch biofilm reactor (IFAS-SBR): Comparison of suspended flocs and attached biofilm. Int. Biodeterior. Biodegradation.

[5] Satyawali Y, Balakrishnan M (2009). Effect of PAC addition on sludge properties in an MBR treating high strength wastewater. Water Res..

[6] Jiang X, Liu G, Wang M, Zheng M (2015). Formation of polychlorinated biphenyls on secondary copper production fly ash: mechanistic aspects and correlation to other persistent organic pollutants. Sci. Rep..

[7] Ahmaruzzaman M (2010). A review on the utilization of fly ash. Prog. Energy Combust. Sci..

[8] Fan H (2018). Effect of the formulation control agent on brightness of modified fly ash and its potential application in papermaking. BioResources..

[9] Gao W, Fatehi P (2018). Fly ash based adsorbent for treating bleaching effluent of kraft pulping process. Sep. Purif. Technol..

[10] Chen X, Si C, Fatehi P (2017). Pretreatment and *in situ* fly ash systems for improving the performance of sequencing batch reactor in treating thermomechanical pulping effluent. ACS Sustain. Chem. Eng..

[11] Payá J (2002). Advantages in the use of fly ashes in cements containing pozzolanic combustion residues: silica fume, sewage sludge ash, spent fluidized bed catalyst and rice husk ash. J. Chem. Technol. Biotechnol..

[12] Ye F, Ye Y, Li Y (2011). Effect of C/N ratio on extracellular polymeric substances (EPS) and physicochemical properties of activated sludge flocs. J. Hazard. Mater..

[13] Zhang P (2014). Composition of EPS fractions from suspended sludge and biofilm and their roles in microbial cell aggregation. Chemosphere.

[14] Ramesh A (2006). Biofouling in membrane bioreactor. Sep. Sci. Technol..

[15] Liu Y, Fang HH (2003). Influences of extracellular polymeric substances (EPS) on flocculation, settling, and dewatering of activated sludge. Crit. Rev. Environ. Sci. Technol..

[16] Sheng GP, Yu HQ, Li XY (2010). Extracellular polymeric substances (EPS) of microbial aggregates in biological wastewater treatment systems: a review. Biotechnol. Adv..

[17] Li XY, Yang SF (2007). Influence of loosely bound extracellular polymeric substances (EPS) on the flocculation, sedimentation and dewaterability of activated sludge. Water Res..

[18] Geyik AG, Çeçen F (2016). Production of protein- and carbohydrate-EPS in activated sludge reactors operated at different carbon to nitrogen ratios. J. Chem. Technol. Biotechnol..

[19] Liao BQ, Allen DG, Droppo IG, Leppard GG, Liss SN (2001). Surface properties of sludge and their role in bioflocculation and settleability. Water Res..

[20] Wang BB (2014). The important implications of particulate substrate in determining the physicochemical characteristics of extracellular polymeric substances (EPS) in activated sludge. Water Res..

[21] Schultz, J. R. & Keinath, T. M. Powdered activated carbon treatment process mechanisms. *JWPCF* **56**, 143–151, http://www.jstor.org/stable/25042186 (1984).

[22] Liao BQ, Lin HJ, Langevin SP, Gao WJ, Leppard GG (2011). Effects of temperature and dissolved oxygen on sludge properties and their role in bioflocculation and settling. Water Res..

[23] Aktaş Ö, Çeçen F (2006). Effect of activation type on bioregeneration of various activated carbons loaded with phenol. J. Chem. Technol. Biotechnol..

[24] Eckenfelder, W. W. Industrial water pollution control (McGraw-Hill, 1989).

[25] Wanner, J. Activated sludge: bulking and foaming control (CRC Press, 2014).

[26] Lin HJ (2009). Sludge properties and their effects on membrane fouling in submerged anaerobic membrane bioreactors (SAnMBRs). Water Res..

[27] Yang SF, Li XY (2009). Influences of extracellular polymeric substances (EPS) on the characteristics of activated sludge under non-steady-state conditions. Process Biochem..

[28] Basuvaraj M, Fein J, Liss SN (2015). Protein and polysaccharide content of tightly and loosely bound extracellular polymeric substances and the development of a granular activated sludge floc. Water Res..

[29] Lin H (2014). A critical review of extracellular polymeric substances (EPSs) in membrane bioreactors: characteristics, roles in membrane fouling and control strategies. J. Membr. Sci. Technol..

[30] Laspidou CS, Rittmann BE (2002). A unified theory for extracellular polymeric substances, soluble microbial products, and active and inert biomass. Water Res..

[31] Jin B, Wilén BM, Lant P (2003). A comprehensive insight into floc characteristics and their impact on compressibility and settleability of activated sludge. Chem. Eng. J..

[32] Characklis, W. G., Turakhia, M. H. & Zelver, N. Transport and interfacial transfer phenomena. *Biofilms* 265–340 (1990).

[33] Sheng GP, Yu HQ, Li XY (2006). Stability of sludge flocs under shear conditions: roles of extracellular polymeric substances (EPS). Biotechnol. Bioeng..

[34] Wilén BM, Jin B, Lant P (2003). The influence of key chemical constituents in activated sludge on surface and flocculating properties. Water Res..

[35] Sponza DT (2004). Properties of four biological flocs as related to settling. J. Environ. Eng..

[36] Ye FX, Shen DS, Feng XS (2004). Anaerobic granule development for removal of pentachlorophenol in an upflow anaerobic sludge blanket (UASB) reactor. Process Biochem..

[37] Zheng Y (2017). Interaction of earthworms-microbe facilitating biofilm dewaterability performance during wasted activated sludge reduction and stabilization. Sci. Total Environ..

[38] Park YS, Kim DS, Park TJ, Song SK (2000). Effect of extracellular polymeric substances (EPS) on the attachment of activated sludge. Bioprocess Eng..

[39] Chen X, Si C, Fatehi P (2018). Enhancement in biological treatment of pulping wastewater by fly ash. Chemosphere.

[40] American Public Health Association. Standard methods for the examination of water and wastewater. Washington, DC (2005).

[41] Cassidy DP, Belia E (2005). Nitrogen and phosphorus removal from an abattoir wastewater in a SBR with aerobic granular sludge. Water Res..

[42] Latif, U. & Dickert, F. L. Biochemical oxygen demand (BOD) in *Environmental analysis by electrochemical sensors and biosensors* (ed. Moretto, L. M. & Kalcher, K.) 729–734, 10.1007/978-1-4939-1301-5_2 (Springer, 2015).

[43] Dai Y (2017). Role of organic compounds from different EPS fractions and their effect on sludge dewaterability by combining anaerobically mesophilic digestion pre-treatment and Fenton’s reagent/lime. Chem. Eng. J..

[44] Lowry OH, Rosebrough NJ, Farr AL, Randall RJ (1951). Protein measurement with the Folin phenol reagent. J. Biol. Chem..

[45] Avella AC, Görner T, de Donato P (2010). The pitfalls of protein quantification in wastewater treatment studies. Sci. Total Environ..

[46] Fatehi P, Gao W, Sun Y, Dashtban M (2016). Acidification of prehydrolysis liquor and spent liquor of neutral sulfite semichemical pulping process. Bioresour. Technol..

[47] Gao W, Han M, Qu X, Xu C, Liao B (2013). Characteristics of wastewater and mixed liquor and their role in membrane fouling. Bioresour. Technol..

[48] Zhang LS, Wu WZ, Wang JL (2007). Immobilization of activated sludge using improved polyvinyl alcohol (PVA) gel. J. Environ. Sci..

[49] Sun Y, Liu Z, Fatehi P (2017). Treating thermomechanical pulping wastewater with biomass-based fly ash: modeling and experimental studies. Sep. Purif. Technol..

